# Identification of Users for a Smoking Cessation Mobile App: Quantitative Study

**DOI:** 10.2196/jmir.7606

**Published:** 2018-04-09

**Authors:** SK Leon Chevalking, Somaya Ben Allouch, Marjolein Brusse-Keizer, Marloes G Postel, Marcel E Pieterse

**Affiliations:** ^1^ Research Group Technology, Health & Care Saxion University of Applied Sciences Enschede Netherlands; ^2^ Medical School Twente Medisch Spectrum Twente Enschede Netherlands; ^3^ Tactus Addiction Treatment University of Twente Enschede Netherlands; ^4^ Centre for eHealth and Well-Being Research Department of Psychology, Health and Technology University of Twente Enschede Netherlands

**Keywords:** mobile applications, telemedicine, mHealth, eHealth, tobacco, smoking cessation, health informatics

## Abstract

**Background:**

The number of mobile apps that support smoking cessation is growing, indicating the potential of the mobile phone as a means to support cessation. Knowledge about the potential end users for cessation apps results in suggestions to target potential user groups in a dissemination strategy, leading to a possible increase in the satisfaction and adherence of cessation apps.

**Objective:**

This study aimed to characterize potential end users for a specific mobile health (mHealth) smoking cessation app.

**Methods:**

A quantitative study was conducted among 955 Dutch smokers and ex-smokers. The respondents were primarily recruited from addiction care facilities and hospitals through Web-based media via websites and forums. The respondents were surveyed on their demographics, smoking behavior, and personal innovativeness. The intention to use and the attitude toward a cessation app were determined on a 5-point Likert scale. To study the association between the characteristics and intention to use and attitude, univariate and multivariate ordinal logistic regression analyses were performed.

**Results:**

The multivariate ordinal logistic regression showed that the number of previous quit attempts (odds ratio [OR] 4.1, 95% CI 2.4-7.0, and OR 3.5, 95% CI 2.0-5.9) and the score on the Fagerstrom Test of Nicotine Dependence (OR 0.8, 95% CI 0.8-0.9, and OR 0.8, 95% CI 0.8-0.9) positively correlates with the intention to use a cessation app and the attitude toward cessation apps, respectively. Personal innovativeness also positively correlates with the intention to use (OR 0.3, 95% CI 0.2-0.4) and the attitude towards (OR 0.2, 95% CI 0.1-0.4) a cessation app. No associations between demographics and the intention to use or the attitude toward using a cessation app were observed.

**Conclusions:**

This study is among the first to show that demographic characteristics such as age and level of education are not associated with the intention to use and the attitude toward using a cessation app when characteristics related specifically to the app, such as nicotine dependency and the number of quit attempts, are present in a multivariate regression model. This study shows that the use of mHealth apps depends on characteristics related to the content of the app rather than general user characteristics.

## Introduction

### Background

Smoking is a serious health problem. The World Health Organization estimates that over 1 billion people worldwide smoke tobacco, and the deaths of 6 million people per year can be attributed to causes linked to smoking [1]. The global prevalence of tobacco use is estimated to be 19%: 1 of 5 people older than 15 years of age uses tobacco, and in developed countries, this statistic is even higher, that is, 1 of 4 people older than 15 years uses tobacco [2]. The prevalence of tobacco use in the Netherlands was estimated to be 26.1% in 2015 among Dutch citizens older than 17 years of age [2].

Attempts to quit smoking are not monitored worldwide by a single organization, but statistics are available for specific nations. In the United States, among current smokers and former smokers, in 2012, an estimated 53% had made a quit attempt longer than 1 day [3]. Estimations from surveys of Dutch smokers [4] show that in 2014, approximately 1 in 3 smokers, accounting for approximately 500,000 smokers, attempted to quit smoking.

Independent quit attempts are unregistered, and because the uptake of interventions (digital and face-to-face) are generally unpredictable [5,6], there is no scientific literature available that relates quit attempts to categories of interventions to show which interventions are popular among smokers. Although there is a worldwide growing uptake of eHealth interventions, objective scientific data about who uses these kinds of interventions and the success of these interventions are almost nonexisting, despite international efforts to gather these data [7-9].

### Mobile Cessation Apps

Recent studies identify a growing body of literature concerning various types of health care apps, resulting in several classifications and taxonomies [10-12]. One of the more recent types of eHealth interventions used for smoking cessation is cessation apps. As Eysenbach [13] noted in 2001, eHealth applies to the dynamic environment in which more is encompassed than just the internet and medicine, which is certainly true for the upcoming mHealth apps that are now part of the broader eHealth environment. There are several mobile phone apps for cessation available to a broad audience; however, results show [8] that most of them are not based on scientific, established guidelines for smoking cessation and do not have a tailored approach meeting individual needs. Due to this gap in the existing literature, the objective of our study was to develop a cessation app based on behavioral strategies and a user-centered approach. In this paper, we report on the characteristics of the potential end users for our mHealth cessation app.

Mobile apps on mobile devices (such as mobile phones and tablets) are fairly common. Research on mobile cessation apps has shown that the number of cessation apps available to consumers and their use are rising steadily [8,9,14,15]. An explanation for this growth can be found in the market potential for cessation apps, as smokers are likely to own a mobile phone, and smokers who intend to quit use their mobile phone more frequently than smokers who have no intention to quit [16].

Studies on cessation apps focus on different outcomes such as app usage [17-19], changes in psychological state related to cessation behavior [20-22], and satisfaction with the treatment with an app [18,20]. To date, effectiveness outcomes have been reported in 3 studies with self-reported abstinence [17,18,20,23], and 1 study protocol with biochemically validated abstinence outcome was found [22]. Thus, although an initial body of research on the use of cessation apps exists, little is known about the factors that influence the use of cessation apps and the characteristics of the users of these mHealth apps.

Studies [18] show that 50% of users found a cessation app useful as a means of support to quit smoking. In addition, participants have been given the choice to use a mobile cessation app to support them, and the result was that when given the choice, 60% of the participants actually used the mobile apps [17]. Both studies [17,18] note that their study populations might not be completely representative of the general smoking population because the study populations included respondents with an affinity for digital smoking support.

### Potential Users

There is little evidence on what population of smokers uses cessation apps and if they share characteristics that can predict mobile cessation app use or explain why such a population is motivated to use cessation apps [7]. The only information available to identify the characteristics of potential users of a smoking cessation app is evidence from the general literature on the use of mobile apps. Research has shown that demographics and personality characteristics are associated with the adoption and use of mobile apps [24-30]. Income and level of education are positively correlated with mobile phone appropriation and adoption, whereas age is negatively correlated with mobile phone appropriation and adoption. However, gender is not correlated with appropriation or adoption [24,27]. We do not know if these findings apply for potential users of mobile cessation apps, which makes it difficult to predict the usefulness of specific mobile app elements, such as a reward system, color schemes, and gamification elements.

Recent findings show that the Big 5 personality traits [31] are related to the adoption of certain categories of mobile apps [26] or the use of a specific app such as Facebook [30,32]. The purpose of this study is to characterize the potential end users for a specific mHealth smoking cessation app, building on the factors known from previous studies on general mobile apps. However, because these are somewhat generic characteristics, for this study, factors related to smoking behavior were added.

These specific characteristics include the level of nicotine dependence, the number of previous quit attempts, and the modes of cessation support used in the past. Such variables have been shown to predict quit attempts in smokers and the likelihood of sustaining abstinence and might therefore also determine the decision to adopt and use a mobile cessation app. In the context of the Integrated Change model (or I-Change Model), these generic and smoking behavior-related characteristics are predisposing factors, which are considered to be distal determinants affecting indirectly behavior through more proximal cognitive determinants such as intention, attitude, and self-efficacy expectancy. Although these proximal determinants are generally considered more powerful predictors of behavior on which the content of an app should be based, distal determinants are particularly useful for defining the segments of a population that should be targeted.

### Research Questions

This study hypothesizes that the adoption of a mobile smoking cessation app is associated with the personal characteristics of smokers and ex-smokers. On the basis of evidence from past research, we will explore the relationship between the intention to use the app and several predisposing factors that are, in nature, demographic (age, gender, educational level, residential area), technology-related (personal innovativeness), or smoking behavior-related (nicotine dependency, number of previous quit attempts). We will also explore the relationship between the attitude toward a mobile smoking cessation app and the aforementioned predisposing factors.

## Methods

### Recruitment and Data Collection

This study used a quantitative approach by using a Web-based questionnaire among smokers and ex-smokers from December 2015 to March 2016. The link to the questionnaire was distributed during the entire period via different social media (Twitter, LinkedIn, WhatsApp, etc), recruiting participants through the digital snowballing method.

Before starting the questionnaire, the respondents read an introductory text on the project and the context wherein the questionnaire was taken. The participants were informed that when they entered their name and email address at the end of the questionnaire, they could be randomly selected to claim a gift card.

The inclusion criteria for this study were that the participants were able to access the Web-based questionnaire and that they were smokers or ex-smokers. Smokers are defined as respondents who smoked regularly at the time they completed the questionnaire. Ex-smokers are defined as respondents who smoked regularly but who have quit. There were no restrictions placed on the quit date. Smokers who are currently attempting to quit are registered as ex-smokers if their quit attempt lasted longer than 24 hours at the time they completed the questionnaire.

### Measures

The questionnaire consisted of 8 constructs, which can be found in Table 1. When available, we used validated scales used in previous research on the adoption of mobile apps or cessation behavior. The complete questionnaire can be found in Multimedia Appendix 1.

### Factor Analysis and Internal Consistency

The dependent variables were used on the scalar level. Factor analysis was used to ensure that the measured scales are unidimensional. The use of factor analysis is applicable, because the sample size is larger than 500 [36]. Additionally, Cronbach alpha was used to determine the internal consistency of the items composing the scales for behavioral intention, attitude, personal innovativeness, and the Fagerstrom Test of Nicotine Dependence.

### Univariate and Multivariate Ordinal Logistic Regression

Due to the ordinal nature of the dependent variables, ordinal logistic regression was used to measure the relationship between the dependent and independent variables [37].The analyses were performed with IBM SPSS statistics for Windows, version 23.0, as the statistical package.

The associations between categorical independent and dependent variables were tested with chi-square tests, followed by a univariate ordinal regression model. Continuous variables were also tested using a univariate ordinal regression model.

Variables associated with *P*<.05 were all entered in a multivariate ordinal regression model. Subsequently, the variables with the highest *P* value were deleted from the model until the model fit decreased significantly.To prevent univariate and multivariate models from becoming unstable or unreliable because of empty cells, categories of categorical variables were omitted or grouped together. In the Results section, these steps are described in detail.

**Table 1:** Summary of study variables. N/A: not available.

Study variables		Item number	Reference
**Independent variables**			
	Current smoking status	1-2	N/A
	Nicotine dependence	3-8	[33]
	Number of quit attempts	9	N/A
	Previous use of digital cessation support	10	N/A
	Personal innovativeness of IT	11-14	[34]
	Demographics	23-26	N/A
**Dependent variables**			
	Behavioral intention to use the cessation app	15-18	[35]
	Attitude toward using the cessation app	19-22	[35]

## Results

### Sample

A total of 955 respondents who met the inclusion criteria started the Web-based survey, and 79.6% (760/955) respondents filled out the complete survey. The questionnaire was constructed such that research variables such as smoking characteristics, the intention to use a mobile cessation app, and attitude toward a mobile cessation app were at the beginning of the questionnaire, whereas demographic information was at the end of the questionnaire, allowing the respondents to be included based on the inclusion criteria, regardless of the completion of their questionnaire. Of the 955 respondents who started the questionnaire, 73.6% (703/955) still smoked, and 26.4% (252/955) respondents had stopped smoking. Analyses with missing values were conducted on a list-wise deletion per analysis. Tables 2 and 3 show the number of respondents per variable.

A total of 898 respondents provided information about their cessation attempt(s): 61.0% (583/955) claimed to have made more than 1 attempt to quit smoking, 24.0% (229/955) made only 1 attempt to quit, and 9.0% (86/955) respondents had not yet made a quit attempt. Crosstabs of cessation attempts with the current smoking status surprisingly revealed that of the 225 respondents who had stopped smoking, 13 respondents claimed to have not made a quit attempt. This could be due to the explanation of the question, which stated that a quit attempt had to be done consciously and should have lasted longer than 24 hours. Because the questionnaire was distributed in a time period that included New Year’s Day, the respondents might have been in the process of their first quit attempt but not longer than 24 hours. Therefore, these respondents are treated as smokers, without a quit attempt. The alternative would be to combine this group of 13 respondents with the 229 respondents who made 1 quit attempt, which would lead to a shift of 1% in the ratios.

When asked about their previous experiences with digital cessation support, 802 respondents provided information. A total of 66.4% (634/955) indicated no previous experience with digital cessation support. The most commonly used digital cessation support was a Web-based self-management program or cessation app, for 13.0% (124/955) respondents, followed by looking up Web-based information about cessation, for 6.0% (57/955) respondents, having digitally contacted a care professional, 1.1% (10/955) respondents, and looking for Web-based peer support (eg, through a Web-based forum), for 0.9% (9 /955) respondents.

### Factor Analysis

Factory analysis was conducted to determine whether the multi-item independent and dependent variables measured with a Likert-scale were unidimensional. Although factor analysis on ordinal variables has risks of over-dimensionalization [38], considering the limited number of items (3-4) and the large number of respondents, it is applied in this study.

All 4 items regarding behavioral intention (n=730) correlated to at least .5, suggesting factorability [39,40]. The Kaiser-Meyer-Olkin measure of sampling adequacy was .76, which is above the suggested value of .6. Bartlett test of sphericity was significant (*P*<.001). Communalities were all above .5. Given these findings, factor analysis was deemed suitable for these 4 items. Principal component analysis (PCA) shows that the largest factor explains 72% of the variance, being the only factor with an initial eigenvalue total higher than 1. All items load onto a single component, with a minimum value of .77. This leads to the conclusion that behavioral intention is a unidimensional scale.

The items regarding attitude (n=730) correlate to at least .7, except item 2 (min. .17). The Kaiser-Meyer-Olkin measure of sampling adequacy was .76, which is above the suggested value of .6. Bartlett test of sphericity was significant (*P*<.001).

**Table 2:** Descriptive characteristics of the study population (N=955).

Variable	Category	Number of participants (%)
Age	Scale	719 (75.3)
Gender	Categorical	719 (75.3)
Educational level	Ordinal	719 (75.3)
Residential area	Categorical	719 (75.3)
Personal innovativeness	Ordinal	857 (89.7)
Nicotine dependence (daytime smoking)	Ordinal	680 (71.2)
Current smoking status	Categorical	898 (94.0)
Number of quit attempts	Ordinal	898 (94.0)
Previous use of digital smoking cessation support	Categorical	802 (84.0)
Behavioral intention to use a mobile smoking cessation app	Ordinal	730 (76.4)
Attitude toward using a mobile smoking cessation app	Ordinal	730 (76.4)

**Table 3:** Descriptive demographic characteristics of the study population (N=955). SD: standard deviation; PhD: Doctor of Philosophy.

Variable		Total
Age, mean in years (SD)		38.0 (13.6)
**Gender, n (%)^a^**		
	Female	470 (49.2)
	Male	245 (25.6)
	Invalid responses	4 (0.4)
**Highest completed educational level, n (%)^a^**		
	Elementary school or secondary education	250 (26.2)
	Vocational degree	220 (23.0)
	Polytechnic education or university of applied science	201 (21.0)
	Scientific degree (Master’s and PhD)	48 (5.0)

^a^Data missing of N=236.

Communalities were all above .8, except for item 2 (.1). These findings suggest that factor analysis is deemed suitable, with the indication that item 2 does not load onto the same factor as the others. PCA confirms this point, showing that the first factor explains 65% of the total variance, being the only factor with an initial eigenvalue total higher than 1. All items load onto a single component, with a minimum value of .90, except item 2 (.34). Item 2 is therefore eliminated from the scale measuring attitude to preserve the unidimensional scale.

The personal innovativeness scale (n=857) consists of 4 items that all correlate to at least.5. The Kaiser-Meyer-Olkin measure of sampling adequacy was .80, which is above the suggested value of.6. Bartlett test of sphericity was significant (*P*<.001). Communalities were all above.5. Factor analysis was deemed suitable based on these findings. PCA shows that the first factor explains 65% of the total variance, being the only factor with an initial eigenvalue total higher than 1. All items load onto a single component, with a minimum value of.76. This leads to the conclusion that personal innovativeness is a unidimensional scale.

Research using confirmatory factor analysis on the Fagerstrom Test of Nicotine Dependence indicates that the scale is best modeled as 2 correlated factors with a cross-loading [39]. These 2 factors are a morning smoking factor and a daytime smoking factor.

We performed exploratory factor analysis and found that the Fagerstrom Test of Nicotine Dependence scale (n=680) consisted of 6 items that loaded onto 2 different factors, with initial eigenvalues of 2.4 and 1.1. The first factor consisted of the following items (the rotated factor loadings are noted in parentheses): *How soon after you wake up do you smoke your first cigarette?* (.37), *Which cigarette would you hate most to give up?* (.60), and *Do you smoke more frequently during the first hours after waking than during the rest of the day?* (.45), resulting in a *morning smoking* factor. The second factor consisted of the following items: *How soon after you wake up do you smoke your first cigarette?* (.72), *On average, how many cigarettes are you currently smoking each day?* (.73), *Do you find it difficult to keep from smoking in places where it is not allowed?* (.39), and *Do you smoke if you are so ill that you are in bed most of the day?* (.50), resulting in a “daytime smoking factor” on which we will conduct our further analysis.

### Internal Consistency

Both dependent variables show good internal consistency, as shown in Table 4. The measure of attitude consists of 3 items, after item 2 was deleted. Both variables are taken as scales in the ordinal logistic regression analyses as follows: the scores for each item are added together, dividing the total score by the number of items and rounding the result to the nearest integer, for example, 1.5 becomes 2, creating a scale with 5 categories.

**Table 4:** Cronbach alpha values for the scale variables.

Variable		Number of respondents	Number of items	Cronbach alpha
**Dependent variables**				
	Behavioral intention to use	730	4	.87
	Attitude toward using	730	3	.91
**Independent variables**				
	Personal innovativeness of Information Technology	857	4	.82
	Morning smoking factor	680	3	.51
	Daytime smoking factor	680	4	.68

The internal consistency of the independent variables of personal innovativeness of Information Technology (IT), morning smoking, and daytime smoking is shown in Table 4. The internal consistency of the personal innovativeness scale is good, whereas the internal consistency of morning smoking is questionable and that of daytime smoking is acceptable. The remainder of the analysis is therefore conducted based on the daytime smoking factor, as this factor best captures nicotine dependency [41,42], and morning smoking has a questionable Cronbach alpha value of .51.

### Univariate Ordinal Logistic Regression

Crosstabs between the dependent and independent variables showed empty cells for the variables of *educational level*, *residential area,* and *previous use of digital cessation support* for both the dependent variables and the personal innovativeness items 1, 3, and 4 on attitude *.* Empty cells in ordinal logistic regression may cause goodness-of-fit predictors to become unreliable. Univariate analysis of single personal innovativeness items on attitude is therefore omitted. The variable *educational level* was reduced to 4 categories to eliminate all empty cells: (1) elementary school and secondary education, (2) vocational degree, (3) polytechnic or university of applied science, and (4) scientific degree or higher.

The variable *residential area* had only 1 empty cell, and the parameter estimates showed no significant results at the level of *P*<.05, making it unnecessary to reduce the number of categories for either the dependent or independent variable. The categories for the variable *previous use of digital cessation support* are grouped into 2 groups: respondents who have used digital cessation support (n=168) and respondents who have not (n=634). Additionally, the categories of the dependent variables were reduced from 5 to 3 to eliminate the remaining empty cells: (1) agree, (2) neutral, and (3) disagree.

The results in Tables 5 and 6 should be interpreted as corresponding to the coding of the questionnaire where favorable outcomes (agree) were coded with low values and undesirable outcomes (disagree) with high values. This means that an odds ratio, OR <1 implies a positive correlation.

Table 5 shows that there is no significant effect for the odds that males have a higher intention to use the cessation app than females. For educational level, there is a significant effect that smokers and ex-smokers with a lower educational level have a higher intention to use the cessation app. For residential area, no significant effects that smokers and ex-smokers living outside the city limits have a higher intention to use the app than smokers and ex-smokers living in a village or in a city were found. The respondents who smoke but have the intention to quit have a higher intention to use the cessation app than ex-smokers. There is no evidence that not having the intention to quit smoking is associated with the intention to use a mobile smoking cessation app.

Table 6 shows that the associations found between the independent variables and attitude are the same as the associations between the independent variables and behavioral intention.

### Scale Ordinal Logistic Regression

As multiple items on the personal innovativeness scale and the Fagerstrom Test of Nicotine Dependence showed significant relationships with the dependent variables, ordinal logistic regression is performed with the scale variables to determine whether the results change.

Crosstabs for the personal innovativeness scale and both behavioral intention and attitude show empty cells at the end categories for the personal innovativeness scale. The personal innovativeness scale is reduced to 3 categories: (1) low personal innovativeness (ranging from 1 to 2.5), (2) average personal innovativeness (ranging from 2.5 to 3.5), and (3) high personal innovativeness (ranging from 3.5 to 5).

### Multivariate Ordinal Logistic Regression

Multivariate ordinal logistic regression is performed with the independent variables *educational level*, *nicotine dependency (daytime smoking)*, *current smoking status*, *and number of quit attempts* and *personal innovativeness* for *behavioral intention* as the dependent variable. *Previous use of digital cessation support,* although significant, is omitted from the multivariate analysis because the outcome measures were adjusted to reduce the number of empty cells. Analysis on the dependent variable of *attitude* includes the same independent variables, excluding *educational level* because of an unacceptable goodness-of-fit.

Multicollinearity tests for both behavioral intention and attitude as the dependent variable show that *current smoking status* has a tolerance of 0.012 (*behavioral intention*) and 0.011 (*attitude*) and variance inflation factor scores >10. The variable is therefore omitted from the multivariate analysis on both dependent variables because its predictability is largely accounted for by the other variables.

**Table 5:** Univariate ordinal regression analyses on behavioral intention. An odds ratio lower than 1 positively correlates with the independent variable.

Variable		Odds ratio (95% CI)
Age (n=715)		1.00 (0.99-1.01)
**Gender (n=715)**		
	Male	0.94 (0.70-1.26)
	Female (base level)	1 (0)
**Educational level^a^ (n=711)**		
	Elementary + secondary education	0.48 (0.30-0.76)
	Vocational degree	0.51 (0.33-0.81)
	Polytechnic + university applied sciences	0.52 (0.33-0.83)
	Scientific education (base level)	1 (0)
**Residential area (n=717)**		
	City	1.73 (0.78-3.80)
	Village	1.66 (0.73-3.76)
	Outside city/village limits (base level)	1 (0)
**Personal innovativeness^a^ (n=730)**		
	High	0.31 (0.21-0.49)
	Moderate	0.47 (0.33-0.67)
	Low (base level)	1 (0)
Nicotine dependency^a^, daytime smoking; 0=low dependency (n=554)		0.89 (0.77-0.89)
**Current smoking status^a^ (n=730)**		
	Smokes but intention to quit	0.21 (0.15-0.29)
	Smokes and no intention to quit^b^	1.29 (0.82-2.03)
	Quit smoking (base level)	1 (0)
**Number of quit attempts^a^ (n=730)**		
	0	3.38 (2.12-5.40)
	1	1.38 (1.00-1.90)
	More than 1 (base level)	1 (0)
**Previous use of digital cessation support^a^ (n=657)**		
	Has experience with digital cessation support; 0=has experience	0.48 (0.33-0.71)
	No experience (base level); 1=no experience	1 (0)

^a^*P* value <.05.

^b^No significant results due to goodness of fit.

**Table 6:** Univariate ordinal regression analyses on attitude. An odds ratio lower than 1 positively correlates with the independent variable.

Variable		Odds ratio (95% CI)
Age (n=715)		1.00 (0.99-1.01)
**Gender (n=715)**		
	Male	0.92 (0.68-1.24)
	Female (base level)	1 (0)
**Educational level^a^ (n=711)**		
	Elementary + secondary education	0.61 (0.38-0.98)
	Vocational degree	0.60 (0.38-0.96)
	Polytechnic + university applied sciences	0.58 (0.36-0.93)
	Scientific education (base level)	1 (0)
**Residential area (n=717)**		
	City	1.24 (0.56-2.75)
	Village	1.33 (0.58-3.06)
	Outside city/village limits (base level)	1 (0)
**Personal innovativeness^b^ (n=730)**		
	High	0.24 (0.16-0.36)
	Moderate	0.44 (0.31-0.63)
	Low (base level)	1 (0)
Nicotine dependency^b^, daytime smoking; 0=low dependency (n=554)		0.82 (0.76-0.89)
**Current smoking status^b^ (n=730)**		
	Smokes but intention to quit	0.23 (0.16-0.32)
	Smokes and no intention to quit^b^	1.28 (0.82-2.00)
	Quit smoking (base level)	1 (0)
**Number of quit attempts^b^ (n=730)**		
	0	3.21 (2.02-5.12)
	1	1.67 (1.20-2.32)
	More than 1 (base level)	1 (0)
**Previous use of digital cessation support^b^ (n=657)**		
	Has experience with digital cessation support	0.31 (0.18-0.51)
	No experience (base level) 1	1 (0)

^a^No significant results due to goodness of fit.

^b^*P* value <.05.

Tables 7 and 8 should be interpreted in the same manner as Tables 5 and 6, with OR<1 meaning a positive correlation with the dependent variable. Table 7 shows the outcome of the ordinal logistic regression of the variables on *behavioral intention*, which included 540 respondents. *Educational level* is the only nonsignificant variable (*P*=.67 for polytechnic or university of applied science, *P*=.262 for vocational degree, and *P*=.157 for elementary school and secondary education, measured against scientific education as the base level).

Table 8 shows the outcome of the regression with *attitude* as the dependent variable (n=554). All independent variables remain as unique predictors of the respondent’s attitude toward the use of a cessation app. The respondents who score high on *personal innovativeness* are the most positive toward the use of a cessation app compared with respondents who score low on *personal innovativeness*. The respondents who have not made a quit attempt are more negative toward the use of a cessation app compared with respondents who have attempted to quit more than once.

**Table 7:** Results of the multivariate ordinal logistic regression on behavioral intention (n=540). An odds ratio lower than 1 positively correlates with the independent variable.

Variable		Odds ratio (95% CI)
**Educational level**		
	Elementary + secondary education	0.64 (0.35-1.19)
	Vocational degree	0.71 (0.39-1.29)
	Polytechnic + university applied sciences	0.57 (0.31-1.04)
	Scientific education (base level)	1 (0)
**Personal innovativeness^a^**		
	High	0.26 (0.16-0.40)
	Moderate	0.40 (0.26-0.61)
	Low (base level)	1 (0)
Nicotine dependency^a^, daytime smoking; 0=low dependency		0.83 (0.77-0.90)
**Number of quit attempts^a^**		
	0	4.07 (2.37-7.01)
	1	1.67 (1.13-2.48)
	More than 1 (base level)	1 (0)

^a^*P* value <.05.

**Table 8:** Results of the multivariate ordinal logistic regression on attitude (n=554). An odds ratio lower than 1 positively correlates with the independent variable.

Variable		Odds ratio (95% CI)
**Personal innovativeness^a^**		
	High	0.23 (0.14-0.36)
	Moderate	0.39 (0.26-0.61)
	Low (base level)	1 (0)
Nicotine dependency^a^, daytime smoking; 0=low dependency		0.84 (0.77-0.91)
**Number of quit attempts^a^**		
	0	3.48 (2.04-5.94)
	1	1.75 (1.18-2.60)
	More than 1 (base level)	1 (0)

^a^*P* value <.05.

## Discussion

### Potential Users of mHealth Cessation Apps

This study presents a conceptualization of the characteristics of potential end users for a specific mHealth cessation app. By providing a comprehensive overview of the characteristics that influence mHealth app acceptance and behavioral intention from a wide variety of disciplines relevant for health technology acceptance behavior, we tried to provide more insight into the specific variables that can play a role in the complex process of peoples’ technology use for health-related reasons.

Our data were based on a large sample of Dutch smokers and ex-smokers (n=955) in which several categories of variables yielded interesting results. First, correlations were found between the *number of quit attempts*, *nicotine dependency*, *previous use of digital cessation support*, and *personal innovativeness* on attitude toward using a cessation app and the intention to use a cessation app. This shows that smokers, with high dependency and experience with failed attempts, are aware of the need to seek support in attempting to quit. First-time quitters lack this awareness and may need to be targeted with more persuasive messages from either human (professional/private) or digital channels to help them become aware of using an mHealth app for smoking cessation.

This study showed that no significant relationship between demographic characteristics and attitude toward or intention to use a cessation app exists. These findings are supported by a similar study [7] that found similar results based on real cessation app data in Australia, the United States, and the United Kingdom.

Second, examining smoking-related characteristics, we found that the total number of quit attempts and the score on the Fagerstrom test for daytime smoking positively correlated with the attitude toward and intention to use a cessation app. Therefore, it is believed that the findings of this study can provide a basis for the further development of a model that may deepen our understanding of the influence of several factors on people’s use of mHealth apps.

### Study Limitations

Despite the results indicating that the number of quit attempts is positively associated with the intention to use and the attitude toward a cessation app, the definition of “quit attempt” in this study, though not uncommon [43,44], is chosen somewhat arbitrarily. First, the respondents were instructed to count a quit attempt when they did not smoke for over 24 hours. Second, the respondents were counted as ex-smokers when they had not smoked within the past 24 hours. Third, 13 respondents out of 225 provided inconsistent data on their current smoking status and number of quit attempts, stating that although they quit smoking, they did not make a quit attempt.

Moreover, due to some limitations of the dataset caused by empty cells in the ordinal regression analysis, certain correlations could not be analyzed. For example, although personal innovativeness positively correlates with intention to use and attitude toward using, whether there is a correlation between personal innovativeness and the previous use of digital support, which could explain the positive correlation with intention to use and attitude toward using, could not be established.

Another study limitation is that the respondents in this study were mostly recruited through Web-based media, such as email and social media. This might have created a sample biased toward the use of digital means, such as a mobile cessation app. The generalizability of the results is therefore limited.

### Conclusions

This study showed that personal factors influence the adoption of a mobile smoking cessation app. Demographics did not seem to correlate with the intention to use or the attitude toward using a mobile smoking cessation app. Personal characteristics related to smoking or quitting behavior as well as personal innovativeness correlated with the adoption of a mobile smoking cessation app. This study provides useful insights into the concept of an mHealth app for smoking cessation from a user perspective. Future research could employ a study to investigate the potential influence that personal characteristics have on the adoption of mHealth apps to increase the satisfaction and effectiveness of mHealth interventions.

## Appendix

Multimedia Appendix 1 Questionnaire.

## Abbreviations

I-Change Model: Integrated Change model
OR: odds ratio
PCA: principal component analysis
