# Supplementary material for: Identification of Users for a Smoking Cessation Mobile App: Quantitative Study
Source: J Med Internet Res. 2018 Apr 9;20(4):e118. doi: 10.2196/jmir.7606 (PMC5913574; doi:10.2196/jmir.7606)
Supplement: Multimedia Appendix 1 [file jmir_v20i4e118_app1.pdf]

Questionnaire “Characteristics of the Target Group”

Tabel 1 Questionnaire

**Nummer**	**variabele_naam**	**Vraag**	**Antwoord(en)**	**Opmerkingen**
1.	rookgedrag_0	Rookt u momenteel?	1. Ja, ik rook en ben van plan om te stoppen<br>2. Ja, ik rook en ben niet van plan om te stoppen<br>3. Nee, ik rookte maar ik ben gestopt<br>4. Nee, ik heb nooit gerookt	
2.	stimulans_0	Welke mensen stimuleren u om te blijven roken?	1. Familie /gezinsleden<br>2. Vrienden /kennissen<br>3. Collega’s<br>4. Anders,nl. …	
3.	rookgedrag_1	Wat rookt u?	1. Sigaretten<br>2. Shag<br>3. Pijptabak<br>4. Sigaren of cigarillo’s<br>5.E-sigaret met nicotine<br>6. E-sigaret zonder nicotine	Meerdere antwoorden mogelijk
4.	ftq_0	Hoe snel na het wakker worden, steekt u uw eerste sigaret op?	1. Minder dan 5 minuten<br>2. 5 tot 30 minuten<br>3.31 tot 60 minuten<br>4. na 60 minuten	
5.	ftq_1	Vindt u het moeilijk om niet te roken op plaatsen waar het verboden is?	1. Ja<br>2. Nee	
6.	ftq_2	Welke sigaret zou u het moeilijkst op kunnen geven?	1. De eerste<br>2. Elke andere	
7.	ftq_3	Hoeveel sigaretten rookt u elke dag?	1. 10 of minder<br>2. 11 tot en met 20<br>3. 21 tot en met 30<br>4. 31 of meer	
8.	ftq_4	Rookt u in de uren na het opstaan meer per uur, dan	1. Ja<br>2. Nee	

		gedurende de rest van de dag?		
9.	ftq_5	Rookt u als u ziek bent en het grootste deel van de dag in bed ligt?	1. Ja<br>2. Nee	
10.	gestopt_0	Hoe lang geleden bent u gestopt met roken? (rond uw antwoord af op hele dagen/maanden/jaren)	…. dagen/maanden/ jaren	Drop-down voor tijdseenheid
11.	eerste_sig_0	Hoe oud was u ongeveer toen u begon met roken?	…. jaar	
12.	stoppoging_0	Hoeveel stoppogingen heeft u in totaal ondernomen? (Een stoppoging is wanneer u bewust probeert te stoppen en dit langer dan 24 uur volhoud)	1. 0<br>2. 1<br>3. Meer dan 1	
13.	stop_hulp_0	Welke hulpmiddelen heeft u weleens gebruikt om te stoppen met roken?	1. Geen hulpmiddelen gebruikt, zelfstandig gestopt<br>2. Niet-roken cursus in de vorm van groepsbegeleiding<br>3. Niet-roken cursus in de vorm van individuele begeleiding<br>4. Nicotinevervangers zoals kauwgom, pleisters, neusspray, zuigtabletten en/of microtabs (tabletje onder de tong)<br>5. E-sigaret met nicotine<br>6. E-sigaret zonder nicotine<br>7. Medicijnen zoals Zyban, Champix en/of Nortrilen<br>8. Telefonische ondersteuning	
14.	stop_hulp_1	Heeft u weleens gebruik gemaakt van digitale middelen om	1. Ja, ik heb met mijn hulpverlener digitaal contact	Meerdere antwoorden mogelijk

		te stoppen met roken?	gehad (bv. e-mail, digitaal behandelplatform)<br>2. Ja, ik heb digitaal contact gezocht met lotgenoten (bv. forum)<br>3. Ja, ik heb op het internet informatie opgezocht over stoppen met roken<br>4. Ja, ik heb gebruik gemaakt van zelfbehandeling via internet (bv. een stoppen-met-roken programma en/of een Stoppen-met-Roken app)<br>5. Nee, ik heb bij het stoppen met roken geen gebruik gemaakt van digitale hulpmiddelen	
15.	stimulans_1	Welke mensen stimuleren u om te stoppen met roken?	1. Familie /gezinsleden<br>2. Vrienden /kennissen<br>3. Collega's<br>4. Anders,nl. …	
16.	omgeving_0	Roken er mensen in uw directe omgeving?	1. Ja<br>2. Nee	
17.	omgeving_1	Welke mensen roken in uw directe omgeving?	1. Familie /gezinsleden<br>2. Vrienden /kennissen<br>3. Collega's<br>4. Anders, nl. …	
18.	pit_1	Als ik over nieuwe technologieën hoor, zoek ik een manier om ermee te experimenteren.	1. Erg mee eens<br>2. Mee eens<br>3. Niet mee eens/mee oneens<br>4. Mee oneens<br>5. Erg mee oneens	
19.	pit_2	In het algemeen ben ik terughoudend in het proberen van nieuwe technologieën. (reversed)	1. Erg mee eens<br>2. Mee eens<br>3. Niet mee eens/mee oneens<br>4. Mee oneens<br>5. Erg mee oneens	
20.	pit_3	In vergelijking met	1. Erg mee eens	

		mijn vrienden en kennissen ben ik meestal de eerste die nieuwe technologieën uitprobeert.	2. Mee eens<br>3. Niet mee eens/mee oneens<br>4. Mee oneens<br>5. Erg mee oneens	
21.	pit_4	Ik vind het leuk om nieuwe technologieën uit te proberen.	1. Erg mee eens<br>2. Mee eens<br>3. Niet mee eens/mee oneens<br>4. Mee oneens<br>5. Erg mee oneens	
Ik zou gebruik kunnen maken van de stoppen-met-roken app …				
22.	self_efficacy_0	… als ik nooit eerder een soortgelijke app gebruikt heb.	1. Erg mee eens<br>2. Mee eens<br>3. Niet mee eens/mee oneens<br>4. Mee oneens<br>5. Erg mee oneens	
23.	self_efficacy_1	… als ik iemand anders de app zie gebruiken voordat ik het zelf ga proberen. (reversed coded)	1. Erg mee eens<br>2. Mee eens<br>3. Niet mee eens/mee oneens<br>4. Mee oneens<br>5. Erg mee oneens	
24.	self_efficacy_2	… als ik iemand om hulp kan vragen wanneer ik vastloop. (reverse coded)	1. Erg mee eens<br>2. Mee eens<br>3. Niet mee eens/mee oneens<br>4. Mee oneens<br>5. Erg mee oneens	
25.	self_efficacy_3	… als iemand anders me helpt met de eerste stappen. (reverse coded)	1. Erg mee eens<br>2. Mee eens<br>3. Niet mee eens/mee oneens<br>4. Mee oneens<br>5. Erg mee oneens	
26.	self_efficacy_4	… als ik alleen de ingebouwde helpfuncties van de app als hulp heb.	1. Erg mee eens<br>2. Mee eens<br>3. Niet mee eens/mee oneens<br>4. Mee oneens<br>5. Erg mee oneens	
27.	self_efficacy_5	… als iemand mij eerst voordoet hoe het moet. (reverse coded)	1. Erg mee eens<br>2. Mee eens<br>3. Niet mee eens/mee oneens	

			4. Mee oneens<br>5. Erg mee oneens	
28.	self_efficacy_6	… als ik hiervoor soortgelijke apps gebruikt heb. (reverse coded)	1. Erg mee eens<br>2. Mee eens<br>3. Niet mee eens/mee oneens<br>4. Mee oneens<br>5. Erg mee oneens	
29.	self_efficacy_7	… als er niemand in de buurt is die mij vertelt wat ik ermee moet doen	1. Erg mee eens<br>2. Mee eens<br>3. Niet mee eens/mee oneens<br>4. Mee oneens<br>5. Erg mee oneens	
30.	perc_useful_0	Door gebruik te maken van de app bij het stoppen met roken, ben ik in staat sneller te stoppen met roken.	1. Erg mee eens<br>2. Mee eens<br>3. Niet mee eens/mee oneens<br>4. Mee oneens<br>5. Erg mee oneens	
31.	perc_useful_1	Door gebruik te maken van de app kan ik beter stoppen met roken	1. Erg mee eens<br>2. Mee eens<br>3. Niet mee eens/mee oneens<br>4. Mee oneens<br>5. Erg mee oneens	
32.	perc_useful_2	Door gebruik te maken van de app zal ik minder roken.	1. Erg mee eens<br>2. Mee eens<br>3. Niet mee eens/mee oneens<br>4. Mee oneens<br>5. Erg mee oneens	
33.	perc_useful_3	Door gebruik te maken van de app vergroot ik mijn effectiviteit om te stoppen met roken.	1. Erg mee eens<br>2. Mee eens<br>3. Niet mee eens/mee oneens<br>4. Mee oneens<br>5. Erg mee oneens	
34.	perc_useful_4	Door gebruik te maken van de app zal het stoppen met roken gemakkelijker zijn.	1. Erg mee eens<br>2. Mee eens<br>3. Niet mee eens/mee oneens<br>4. Mee oneens<br>5. Erg mee oneens	
35.	perc_useful_5	Ik zou een app nuttig vinden bij het stoppen met roken.	1. Erg mee eens<br>2. Mee eens<br>3. Niet mee eens/mee	

			oneens<br>4. Mee oneens<br>5. Erg mee oneens	
36.	perc_ease_use_0	Leren om de app te gebruiken zal gemakkelijk voor mij zijn	1. Erg mee eens<br>2. Mee eens<br>3. Niet mee eens/mee oneens<br>4. Mee oneens<br>5. Erg mee oneens	
37.	perc_ease_use_1	Ik zal het gemakkelijk vinden om de app te laten doen wat ik wil.	1. Erg mee eens<br>2. Mee eens<br>3. Niet mee eens/mee oneens<br>4. Mee oneens<br>5. Erg mee oneens	
38.	perc_ease_use_2	Mijn interactie met de app zal duidelijk en begrijpelijk zijn.	1. Erg mee eens<br>2. Mee eens<br>3. Niet mee eens/mee oneens<br>4. Mee oneens<br>5. Erg mee oneens	
39.	perc_ease_use_3	Ik zal de app flexibel vinden om met mensen te communiceren en interacties mee te houden.	1. Erg mee eens<br>2. Mee eens<br>3. Niet mee eens/mee oneens<br>4. Mee oneens<br>5. Erg mee oneens	
40.	perc_ease_use_4	Het zal gemakkelijk voor mij zijn om handig te worden in het gebruik van de app.	1. Erg mee eens<br>2. Mee eens<br>3. Niet mee eens/mee oneens<br>4. Mee oneens<br>5. Erg mee oneens	
41.	perc_ease_use_5	Ik zal de app gemakkelijk te gebruiken vinden.	1. Erg mee eens<br>2. Mee eens<br>3. Niet mee eens/mee oneens<br>4. Mee oneens<br>5. Erg mee oneens	
42.	behavioral_int_0	Stel dat ik de app heb, dan ben ik van plan de app te gebruiken.	1. Erg mee eens<br>2. Mee eens<br>3. Niet mee eens/mee oneens<br>4. Mee oneens<br>5. Erg mee oneens	
43.	behavioral_int_1	Wanneer mogelijk, ben ik van plan de app te gebruiken bij	1. Erg mee eens<br>2. Mee eens<br>3. Niet mee	

		het stoppen met roken.	eens/mee oneens<br>4. Mee oneens<br>5. Erg mee oneens	
44.	behavioral_int_2	Voor zover mogelijk, zal ik de app gebruiken voor verschillende dingen.	1. Erg mee eens<br>2. Mee eens<br>3. Niet mee eens/mee oneens<br>4. Mee oneens<br>5. Erg mee oneens	
45.	behavioral_int_3	Ik ben van plan het gebruik van de app in de toekomst te verhogen.	1. Erg mee eens<br>2. Mee eens<br>3. Niet mee eens/mee oneens<br>4. Mee oneens<br>5. Erg mee oneens	
46.	att_app_0	Gebruik maken van de app om te stoppen met roken zou voor mij een goed idee zijn.	1. Erg mee eens<br>2. Mee eens<br>3. Niet mee eens/mee oneens<br>4. Mee oneens<br>5. Erg mee oneens	
47.	att_app_1	Gebruik maken van de app tijdens het stoppen met roken is onplezierig voor mij. (reverse coded)	1. Erg mee eens<br>2. Mee eens<br>3. Niet mee eens/mee oneens<br>4. Mee oneens<br>5. Erg mee oneens	
48.	att_app_2	Gebruik maken van de app is voor mij bevorderlijk voor het stoppen met roken.	1. Erg mee eens<br>2. Mee eens<br>3. Niet mee eens/mee oneens<br>4. Mee oneens<br>5. Erg mee oneens	
49.	att_app_3	Ik zou het prettig vinden de app te gebruiken om te stoppen met roken.	1. Erg mee eens<br>2. Mee eens<br>3. Niet mee eens/mee oneens<br>4. Mee oneens<br>5. Erg mee oneens	
50.	compatability_0	Het gebruik van de app sluit aan bij alle aspecten van mijn stoppen met roken proces.	1. Erg mee eens<br>2. Mee eens<br>3. Niet mee eens/mee oneens<br>4. Mee oneens<br>5. Erg mee oneens	
51.	compatability_1	Gebruik maken van de app past bij mijn	1. Erg mee eens<br>2. Mee eens	

		levensstijl.	3. Niet mee eens/mee oneens<br>4. Mee oneens<br>5. Erg mee oneens	
52.	compatability_2	Ik denk dat gebruik maken van de app goed past bij de manier waarop ik wil stoppen met roken.	1. Erg mee eens<br>2. Mee eens<br>3. Niet mee eens/mee oneens<br>4. Mee oneens<br>5. Erg mee oneens	
53.	individual_0	Het gebruik van de app is afhankelijk van iemands kennis van ICT-toepassingen.	1. Erg mee eens<br>2. Mee eens<br>3. Niet mee eens/mee oneens<br>4. Mee oneens<br>5. Erg mee oneens	
54.	individual_1	Het gebruik van de app is afhankelijk van de leeftijd van de gebruiker.	1. Erg mee eens<br>2. Mee eens<br>3. Niet mee eens/mee oneens<br>4. Mee oneens<br>5. Erg mee oneens	
55.	individual_2	Het gebruik van de app is afhankelijk van iemands ervaring met relevante andere apps.	1. Erg mee eens<br>2. Mee eens<br>3. Niet mee eens/mee oneens<br>4. Mee oneens<br>5. Erg mee oneens	
56.	individual_3	Het gebruik van de app is afhankelijk van persoonlijke eigenschappen van de gebruiker.	1. Erg mee eens<br>2. Mee eens<br>3. Niet mee eens/mee oneens<br>4. Mee oneens<br>5. Erg mee oneens	
57.	geslacht_0	Wat is uw geslacht?	1. Man<br>2. Vrouw<br>3. Anders…	
58.	leeftijd_0	Wat is uw leeftijd?	…. jaar	
59.	nationaliteit_0	Wat is uw nationaliteit?	*Open antwoord*	
60.	opleiding_0	Wat is uw hoogst afgeronde opleiding?	1. Basisonderwijs<br>2. LBO/Mavo/VMBO<br>3. Havo<br>4. VWO<br>5. MBO<br>6. HBO<br>7. Post-HBO (HBO-master,	

			premaster, HBO+)<br>8. WO<br>9. Post-WO (doctoraal, PhD)<br>10. Anders…	
61.	arbeid_uur_0	Hoeveel uur per week zet u zich in voor betaald werk, vrijwilligerswerk, mantelzorg of soortgelijke activiteiten?	… uur per week	
62.	woonomg_0	Wat is uw woonomgeving?	1. Ik woon in een stad<br>2. Ik woon in een dorp<br>3. Ik woon buiten de grenzen van een stad of dorp<br>4. Anders …	
